# Phase II study of vemurafenib followed by ipilimumab in patients with previously untreated *BRAF*-mutated metastatic melanoma

**DOI:** 10.1186/s40425-016-0148-7

**Published:** 2016-08-16

**Authors:** Asim Amin, David H. Lawson, April K.S. Salama, Henry B. Koon, Troy Guthrie, Sajeve S. Thomas, Steven J. O’Day, Montaser F. Shaheen, Bin Zhang, Stephen Francis, F. Stephen Hodi

**Affiliations:** 1 Levine Cancer Institute, Carolinas Healthcare System, Medical Oncology, 1021 Morehead Medical Drive, Charlotte, NC 28204 USA; 2 Winship Cancer Institute of Emory University, Atlanta, GA USA; 3 Duke Cancer Institute, Durham, NC USA; 4 Case Comprehensive Cancer Center, Case Western Reserve University, Cleveland, OH USA; 5 Baptist Cancer Institute, Jacksonville, FL USA; 6 MD Anderson Cancer Center Orlando, Orlando, FL USA; 7 John Wayne Cancer Institute at Providence Saint John’s Health Center, Santa Monica, CA USA; 8 University of New Mexico Cancer Center, Albuquerque, NM USA; 9 Bristol-Myers Squibb, Princeton, NJ USA; 10 Dana-Farber Cancer Institute, Boston, MA USA; 11 Current Address: KBP BioSciences, Princeton, NJ USA

**Keywords:** Vemurafenib, Ipilimumab, Melanoma, CTLA-4, Immune checkpoint inhibitor, BRAF inhibitor, Immunotherapy, Targeted agent

## Abstract

**Background:**

Ipilimumab (IPI), an anti-CTLA-4 antibody, and vemurafenib (VEM), a BRAF inhibitor, have distinct mechanisms of action and shared toxicities (e.g., skin, gastrointestinal [GI] and hepatobiliary disorders) that may preclude concomitant administration. Concurrent administration of IPI and VEM previously showed significant dose-limiting hepatotoxicity in advanced melanoma. This single-arm, open-label, phase II study evaluated a sequencing strategy with these two agents in previously untreated patients with *BRAF*-mutated advanced melanoma.

**Methods:**

This study was divided into two parts. During Part 1 (VEM1-IPI), patients received VEM 960 mg twice daily for 6 weeks followed by IPI 10 mg/kg every 3 weeks for 4 doses (induction), then every 12 weeks (maintenance) beginning at week 24 until disease progression or unacceptable toxicity. During Part 2 (VEM2), patients who progressed after IPI received VEM at their previously tolerated dose. The primary objective was to estimate the incidence of grade 3/4 drug-related skin adverse events (AEs) during VEM1-IPI.

**Results:**

All patients who were initially treated with VEM (*n* = 46) received IPI induction therapy; 8 received IPI maintenance and 19 were treated during VEM2. During VEM1-IPI, the incidence of grade 3/4 drug-related AEs associated with the skin, GI tract, and hepatobiliary system was 32.6 %, 21.7 %, and 4.3 %, respectively. There were no drug-related deaths. At a median follow-up of 15.3 months, median overall survival was 18.5 months. Median progression-free survival was 4.5 months.

**Conclusions:**

VEM (960 mg twice daily for 6 weeks) followed by IPI 10 mg/kg has a manageable safety profile. The benefits/risks of BRAF inhibitors followed by immunotherapy should be evaluated further in light of continuing developments in treatment options for metastatic melanoma.

**Trial registration:**

ClinicalTrials.gov identifier: NCT01673854 (CA184-240) Registered 24 August 2012

## Background

The treatment landscape for metastatic melanoma has shifted dramatically in the past five years with the introduction of immune checkpoint inhibitors and targeted agents. In 2011, the anti-CTLA-4 antibody YERVOY® (ipilimumab, IPI; Bristol-Myers Squibb, New York, NY, USA) and BRAF inhibitor Zelboraf® (vemurafenib, VEM; Genentech, San Francisco, CA, USA) were approved for the treatment of metastatic melanoma, marking the first immune checkpoint inhibitor and the first targeted agent, respectively, to be approved for this indication.

The dynamics and durability of response observed with IPI and VEM differ markedly. IPI has demonstrated a durable survival benefit in ~20 % of patients, ~10 years from the initial treatment in a proportion of patients; however, IPI-induced tumor responses often require time to reach their full potential [1–5]. VEM can induce rapid and substantial responses in approximately 50 % of patients with advanced, *BRAF*-mutated melanoma [6, 7], but in most cases responses are not durable, as tumors develop resistance to BRAF inhibition due to activation of alternate signaling pathways [8, 9]. Patients may experience rapid disease progression once tumors bypass BRAF inhibition, often not allowing time for other therapeutic options to be considered.

Several lines of evidence provide a rationale for combining an immune checkpoint inhibitor with a targeted agent to optimize patient outcomes (rapid response with VEM, and durability of response with IPI), while minimizing toxicity [10]. Tumor cell death mediated by inhibition of the MAPK pathway may lead to increased antigen presentation or cross-presentation to tumor-specific T cells [11]. Inhibition of the MAPK pathway has been shown to increase the expression of melanocyte differentiation antigens in melanoma cell lines and fresh tumor digests, conferring enhanced antigen-specific recognition by T lymphocytes [12]. In co-cultures of melanoma cell lines and human monocyte-derived dendritic cells, inhibition of BRAF and MEK restores compromised dendritic cell function [13].

Concurrent administration of VEM and IPI was evaluated in a phase I study of patients with *BRAF*-mutated metastatic melanoma [14]. Patients in the first cohort (*n* = 6) received 960 mg VEM twice daily for 4 weeks, before starting concurrent IPI 3 mg/kg given every 3 weeks for four infusions; in the second cohort (*n* = 6), the dose of VEM was reduced to 720 mg twice daily. Four of six patients in the first cohort experienced grade 3 elevations in aminotransferase levels after the first infusion of VEM plus IPI. Among the first four patients treated in the second cohort, two patients experienced grade 3 elevations in aminotransferase levels after starting IPI. Given the hepatotoxicity observed with VEM plus IPI, the remaining two patients in the second cohort received VEM alone and the study was closed to further patient accrual. All hepatic adverse events (AEs) were asymptomatic and reversible with discontinuation of study therapy or administration of glucocorticoids. Two of six patients in the first cohort experienced grade 3 rash with VEM plus IPI.

The current study was designed to evaluate a sequencing strategy (VEM followed by IPI), in order to avoid the toxicities observed with concurrent administration. The rationale for this treatment sequence was that it could potentially delay or prevent the emergence of BRAF inhibitor resistance by initiating IPI before disease progression, and to optimize a potential immune-synergy by initiating IPI soon after BRAF-inhibitor induced T-cell infiltration.

This report focuses on patients who were treated with VEM followed by IPI during Part 1 of the study (VEM1-IPI). Data collected during Part 2 (subsequent VEM re-treatment, VEM2) are summarized in brief. Grade 3/4 skin AEs were the most frequent severe common toxicities shared by the two agents and the incidence of these events was therefore chosen as the primary endpoint. Grade 3/4 gastrointestinal and hepatobiliary AEs were evaluated as secondary endpoints.

## Methods

### Patients

Eligible patients had histologically confirmed unresectable stage III or IV malignant melanoma that harbored a BRAF V600 mutant. Patients had to be at least 18 years old with measurable disease and an Eastern Cooperative Oncology Group (ECOG) performance status of 0 or 1. Prior systemic treatment for melanoma was not permitted. Patients with primary ocular melanoma, active autoimmune diseases, or symptomatic brain metastases requiring corticosteroid treatment were ineligible.

All patients provided written informed consent. The protocol was approved by the institutional review boards or ethics committees of the participating sites. The study was conducted in accordance with declaration of Helsinki with good clinical practice as defined by the International Conference on Harmonization.

### Study design and treatment

**Fig. 1:**
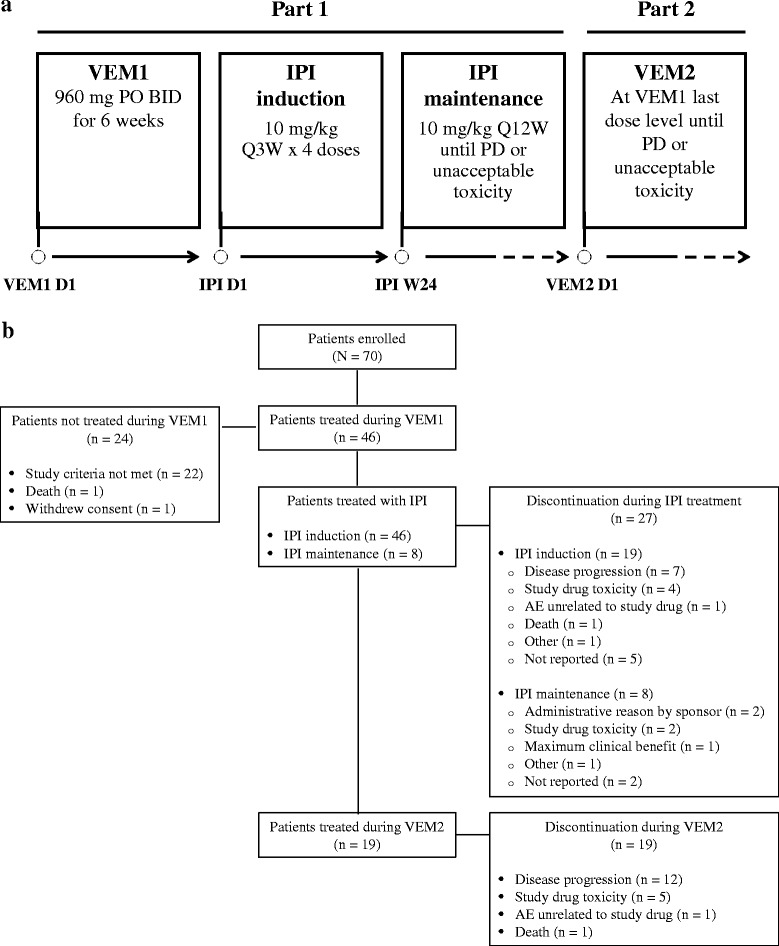
This study was divided into two parts: VEM1-IPI and VEM2. During VEM1-IPI, patients received VEM 960 mg twice daily for 6 weeks followed by IPI 10 mg/kg induction and maintenance therapy. During VEM2, patients who progressed after IPI received VEM at their previously tolerated dose (**a**). Among 70 patients who enrolled in this study, 46 were treated during VEM1 and continued to IPI induction. Eight patients received IPI maintenance therapy. Nineteen patients were treated during VEM2. Reasons for discontinuation of study drug are provided (**b**). AE = adverse event; BID = twice daily; IPI = ipilimumab; PO = by mouth; PD = progressive disease; Q3W = every 3 weeks; Q12W = every 12 weeks; VEM = vemurafenib

This phase II, single-arm, open-label study (CA184-240, ClinicalTrials.gov identifier: NCT01673854) was carried out in two parts (Fig. 1a). During Part 1 (VEM1-IPI), patients received VEM 960 mg orally twice daily for 6 weeks. After a washout period of 3 to 10 days, patients were initiated on IPI induction at 10 mg/kg every 3 weeks for 4 doses. At week 24, patients received IPI maintenance at the dose of 10 mg/kg every 12 weeks until disease progression by modified World Health Organization (mWHO) criteria or unacceptable toxicity for a maximum treatment period of 3 years from the first dose. A higher than approved dose of IPI for advanced melanoma was selected for this study based on clinical data showing a greater mean rate of increase in absolute lymphocyte count and improved efficacy measures (e.g., BOR) with IPI 10 versus 3 mg/kg [15].

During Part 2 (VEM2), patients without progression or unacceptable toxicity to VEM during Part 1 (VEM1-IPI) and who had discontinued IPI were re-treated with VEM at their previously tolerated dose. A minimum of 1-month washout period between IPI and treatment in VEM2 was required for patients who stopped IPI because of toxicity or reasons other than progression. Patients who proceeded to VEM2 after progression on IPI maintenance did not have any minimum washout requirement before re-initiating VEM. Treatment was continued until disease progression by WHO criteria or unacceptable toxicity. Tumor response was assessed by mWHO criteria after completion of VEM1-IPI induction and then every 12 weeks.

### Study objectives

The primary objective was to estimate the incidence of grade 3/4 drug-related skin AEs during VEM1-IPI. Secondary objectives were to estimate the rates of grade 3/4 drug-related gastrointestinal (GI) and hepatobiliary AEs during VEM1-IPI. For both the primary and secondary objectives, analyses included events that occurred from the first dose of VEM1 to the last dose of IPI plus 90 days or to the first dose of VEM2, whichever occurred first. Worst grade drug-related skin, GI, and hepatobiliary AEs were taken into account.

Exploratory objectives included progression-free survival (PFS), best overall response (BOR), objective response rate (ORR), duration of response (DOR), duration of stable disease (DOSD), and safety during VEM1-IPI. PFS was defined as the time between the first dose date of VEM1 and the date of progressive disease by mWHO criteria or death, whichever occurred first. BOR analysis was defined over the VEM1-IPI period as a whole and compared the on-study tumor burden to the study baseline tumor assessment. ORR was defined as the number of patients who achieved a BOR of complete response (CR) or partial response (PR) divided by the total number of IPI-treated patients. DOR was assessed in patients who achieved a BOR of CR or PR, and was defined as the time between the date of confirmed response and the date of progressive disease or death, whichever occurred first. DOSD was defined as the time between the date of the first evaluable tumor assessment with at least SD and the date of progressive disease or death, whichever occurred first. Exploratory safety endpoints included AEs, serious adverse events (SAEs), drug-related AEs, and AEs leading to discontinuation. All AEs were coded using Medical Dictionary for Regulatory Activities (MedDRA) system organ classes and preferred terms, and were graded using the National Cancer Institute Common Terminology Criteria for Adverse Events (NCI CTCAE v3.0).

Although this study was primarily focused on outcomes during VEM1-IPI, additional data during subsequent VEM re-treatment (VEM2) was also collected. All safety analyses performed during VEM1-IPI were also performed during VEM2, and included events from the first dose date of VEM2 to the last dose of VEM2 plus 14 days. ORR during VEM2 was also evaluated.

All treated patients were followed every 12 weeks for survival.

### Statistical analyses

The sample size for this study was not based on power calculation. It was estimated that approximately 45 eligible patients would be treated during VEM1. Allowing for a 10 % drop-out prior to IPI treatment, the number of patients with IPI was expected to be approximately 40. Assuming that the incidence of high-grade skin AEs was approximately 10 %, a sample size of 40 treated patients would provide two-sided exact 95 % confidence intervals (CIs) of 2.8 % to 23.7 %. Time-to-event endpoints (PFS, DOR, DOSD, and OS) were estimated using the Kaplan-Meier (KM) method. The estimate of median and two-sided 95 % CIs was calculated by the Brookmeyer and Crowley method. KM estimates of PFS rates and associated two-sided log-log transformation 95 % CIs were calculated at multiple time points, including 6 months and 1 year. For ORR, exact two-sided 95 % CIs were computed using the Clopper and Pearson method. For primary and secondary safety endpoints, exact two-sided 95 % CIs and point estimates were determined.

## Results

### Patients

Seventy patients were enrolled between October 2012 and July 2014, and 46 were treated during VEM1-IPI (Fig. 1b). Patient demographic and baseline clinical characteristics during VEM1-IPI are summarized in Table 1. The mean age of patients was 55.0 years, and 80.4 % were male. Most patients (76.1 %) had an ECOG performance status of 0. At study entry, most patients had stage IV disease (82.6 %), M1c (52.2 %), and at least 5 disease sites involved (54.3 %).

**Table 1:** Patient demographic and baseline clinical characteristics

Characteristic	VEM1-IPI (*n* = 46)
Age, years	
Mean (SD)	55.0 (14.20)
Gender, n (%)	
Male	37 (80.4)
ECOG performance status, n (%)	
0	35 (76.1)
1	11 (23.9)
Disease stage at study entry, n (%)	
III	8 (17.4)
IV	38 (82.6)
M-stage at study entry, n (%)	
M0	6 (13.0)
M1a	8 (17.4)
M1b	8 (17.4)
M1c	24 (52.2)
Number of disease sites, n (%)	
1	2 (4.3)
2	8 (17.4)
3	7 (15.2)
4	4 (8.7)
≥ 5	25 (54.3)

*ECOG* Eastern Cooperative Oncology Group, *IPI* ipilimumab, *SD* standard deviation, *VEM* vemurafenib

### Drug exposure

All 46 patients treated with VEM during VEM1 continued to IPI induction (Fig. 1b). The median number of doses administered during IPI induction was 4; 24 patients (52.2 %) received all 4 doses, 8 (17.4 %) received 3 doses, 8 (17.4 %) received 2 doses, and 6 (13.0 %) received 1 dose. Eight patients (17.4 %) received IPI maintenance therapy. The median number of doses administered during IPI maintenance was 3; 1 patient each received 5, 6, or 7 doses; 3 patients received 3 doses and 2 patients received 1 dose. Ten patients (21.7 %) required at least one IPI dose delay, and 6 (13.0 %) needed at least one IPI infusion interruption. At the time of this analysis, all 46 patients had discontinued study treatment: 27 during IPI treatment and 19 during VEM2 treatment (Fig. 1b). Reasons for discontinuation of study drugs are provided in Fig. 1b.

### Safety

During VEM1-IPI, 15 of 46 patients (32.6 %) experienced 1 or more grade 3/4 drug-related skin AEs (e.g., rash, erythema, pruritus) (Table 2). Grade 3/4 drug-related GI AEs occurred in 10 of 46 patients (21.7 %); diarrhea was the most common (*n* = 5, 10.9 %). Only 2 of 46 patients (4.3 %) experienced grade 3/4 drug-related hepatobiliary toxicity: hepatitis (*n* = 1, 2.2 %) and hyperbilirubinemia (*n* = 1, 2.2 %).

**Table 2:** Drug-related grade 3/4 skin, gastrointestinal, and hepatobiliary AEs during VEM1-IPI by investigator-reported preferred term

AE Organ Category, n (%)^a^	VEM1-IPI (*n* = 46)
Skin	15 (32.6)
Rash	9 (19.6)
Erythema	2 (4.3)
Exfoliative rash	2 (4.3)
Pruritus	2 (4.3)
Rash generalized	2 (4.3)
Rash maculo-papular	1 (2.2)
Gastrointestinal disorders	10 (21.7)
Diarrhea	5 (10.9)
Colitis	2 (4.3)
Nausea	2 (4.3)
Abdominal pain	1 (2.2)
Autoimmune colitis	1 (2.2)
Vomiting	1 (2.2)
Hepatobiliary disorders	2 (4.3)
Hepatitis	1 (2.2)
Hyperbilirubinemia	1 (2.2)

*AE* adverse event, *IPI* ipilimumab, *VEM* vemurafenib

^a^Patients may have experienced more than 1 event

Overall during VEM1-IPI, 43 of 46 (93.5 %) patients reported a drug-related AE of any grade (Table 3). All 43 patients had 1 or more drug-related AEs that were consistent with an immune phenomenon. Grade 3/4 drug-related AEs were reported in 30 patients (65.2 %). Drug-related serious adverse events (SAEs) of any grade were noted in 18 patients (39.1 %). The only grade 3/4 drug-related SAE observed in 3 or more patients was squamous cell carcinoma (*n* = 3, grade 3 events).

**Table 3:** Drug-related AEs during VEM1-IPI

Event, *n* (%)^a^	VEM1-IPI (n = 46)		
	Any Grade	Grade 3	Grade 4
Any drug-related AE	43 (93.5)	27 (58.7)	3 (6.5)
AEs occurring in ≥ 3 patients^b^			
Rash	28 (60.9)	9 (19.6)	0 (0)
Diarrhea	17 (37.0)	5 (10.9)	0 (0)
AST increased	9 (19.6)	3 (6.5)	1 (2.2)
ALT increased	8 (17.4)	4 (8.7)	0 (0)
Squamous cell carcinoma^c^	3 (6.5)	3 (6.5)	0 (0)
Any drug-related serious AEs	18 (39.1)	15 (32.6)	2 (4.3)
AEs leading to discontinuation of treatment	16 (34.8)	9 (19.6)	1 (2.2)

*AE* adverse event, *ALT* alanine aminotransferase, *AST* aspartate aminotransferase, *IPI* ipilimumab, *VEM* vemurafenib

^a^Patients may have experienced more than 1 event

^b^Only toxicities that reached Grade 3/4 in severity in ≥ 3 patients are presented

^c^Squamous cell carcinoma was classified as a serious adverse event (SAE) and was the only SAE that was observed in 3 or more patients (*n* = 3, grade 3 events)

Drug-related AEs of any grade leading to discontinuation of treatment during VEM1-IPI were most frequently seen as GI disorders (21.7 %), including colitis and diarrhea (6.5 % each), nausea and vomiting (4.3 % each); all other system-organ events leading to discontinuation were each reported in only 1 patient.

During VEM2, drug-related AEs of any grade were observed in 10 of 19 patients (52.6 %). Five patients (26.3 %) experienced a grade 3 drug-related AE during VEM2.

Twenty-four patients (52.2 %) died during this study due to disease (*n* = 22) or other/unknown factors (*n* = 2). There were no drug-related deaths.

### Efficacy

**Fig. 2:**
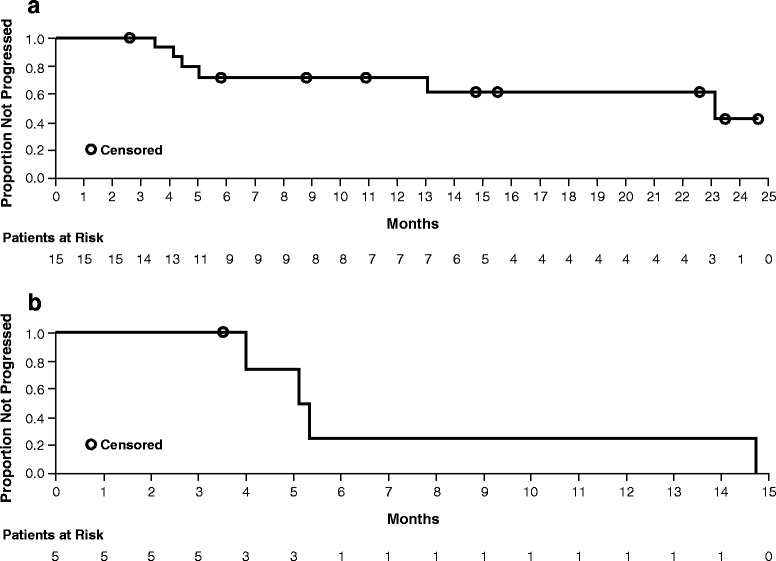
Kaplan-Meier curves for DOR (**a**) and DOSD (**b**) during VEM1-IPI. The median DOR was 23.1 months (95 % CI: 5.03–not evaluable), and the median DOSD was 5.2 months (95 % CI: 3.98–14.75)

**Fig. 3:**
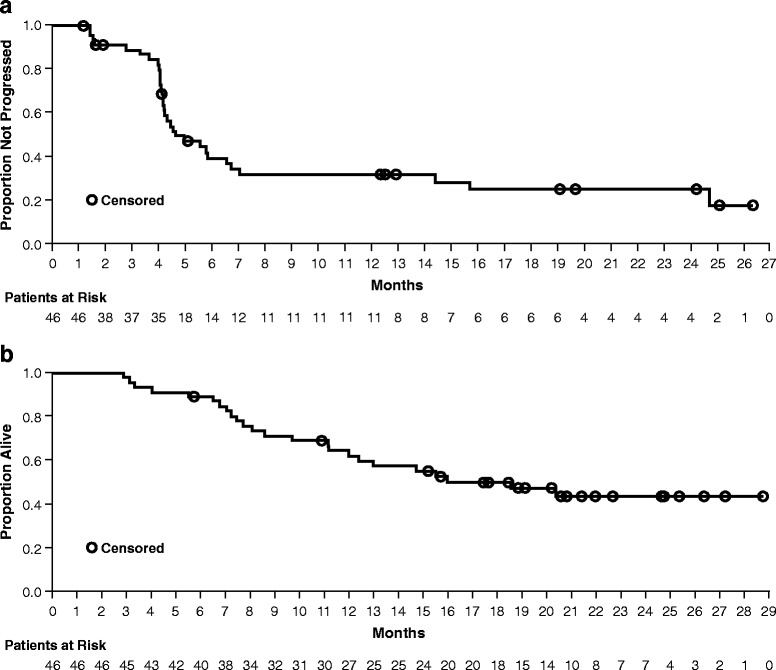
Kaplan-Meier curves for PFS during VEM1-IPI (**a**) and OS (**b**). The median PFS was 4.5 months (95 % CI: 4.17–6.57). At a median follow-up of 15.3 months, the median OS was 18.5 months (95 % CI: 11.96–not evaluable)

During VEM1-IPI, the BOR rate was 32.6 % (Table 4). The median duration of response was 23.1 months (95 % CI: 5.03–not evaluable), and the median duration of stable disease was 5.2 months (95 % CI: 3.98–14.75) (Table 4, Fig. 2). The BOR rate during VEM2 was 36.8 %. The median PFS during VEM1-IPI was 4.5 months (95 % CI: 4.17–6.57) (Fig. 3a). At a median follow-up of 15.3 months, the median OS was 18.5 months (95 % CI: 11.96–not evaluable) (Fig. 3b).

**Table 4:** Efficacy results during VEM1-IPI

	Result
Best overall response, n (%)	
Complete response	2 (4.3)
Partial response	13 (28.3)
Stable disease	5 (10.9)
Progressive disease	11 (23.9)
Unknown^a^	15 (32.6)
Best overall response rate, % (95 % CI)	32.6 (19.5–48.0)
Median duration of response, mo (95 % CI)	23.1 (5.03–NE)
Median duration of stable disease, mo (95 % CI)	5.2 (3.98–14.75)

*CI* confidence interval, *IPI* ipilimumab, mo month, *NE* not evaluable, *VEM* vemurafenib

^a^Response could not be determined due to missing assessment, image quality, etc

## Discussion

This phase II, single-arm, open-label study showed that VEM (960 mg twice daily for 6 weeks) followed by IPI 10 mg/kg can be administered safely in patients with previously untreated *BRAF*-mutated metastatic melanoma. Although 65.2 % of the patients experienced a grade 3/4 drug-related AE, no drug-related deaths occurred. During VEM1-IPI, grade 3/4 drug-related skin AEs were observed in 32.6 % of patients, which is higher than that reported with IPI 10 mg/kg alone (grade 3/4 immune-related skin AEs: 4.2 % in the phase II study CA184-022 and 3.2 % in the phase II study CA184-008) or VEM monotherapy (grade 3 rash: 8 % in the phase III study BRIM-3) [6, 15, 16]. Interestingly, the incidence of grade 3 squamous cell carcinoma in this study (6.5 % based on NCI CTCAE v3.0) was lower than that reported with VEM monotherapy (12 % based on NCI CTCAE v4.0), which is possibly due to the shorter duration of VEM therapy (6 weeks) in our study [6]. Alternatively, this may suggest a role for the immune response in the development of secondary squamous cell carcinomas in light of the possible etiologies of their development via *HRAS* mutations in keratinocytes [17]. The incidence of drug-related grade 3/4 diarrhea (10.9 %) and colitis (4.3 %) reported here is similar to that observed with IPI 10 mg/kg alone (CA184-022: 14.1 % and 2.8 %, respectively) [18]. In contrast to the high hepatotoxicity noted with concurrent administration of VEM and IPI (4 of 6 patients [66.7 %] treated with VEM 960 mg/kg in cohort 1) [14], only 2 of 46 patients (4.3 %) experienced grade 3/4 hepatotoxicity with this VEM/IPI sequencing regimen (Table 2), suggesting that sequential administration of VEM and IPI results in a better toxicity profile than concurrent administration.

The BOR observed during VEM1-IPI was 32.6 %, which is higher than that reported with IPI 10 mg/kg alone (11.1 % in CA184-022 and 5.8 % in CA184-008) [15, 16], and lower than that reported with VEM monotherapy (ORR: 57 % in BRIM-3) [19]. The response observed and reported in this study was captured at the end of completion of 4 doses of induction therapy with IPI and therefore does not necessarily reflect best response to VEM monotherapy at the end of the initial 6-week treatment. Some of the patients with rapidly progressive disease could potentially have had an increase in their tumor burden while receiving IPI prior to the first response evaluation of the study. During VEM1-IPI, the median duration of response was 23.1 months, and the median duration of stable disease was 5.2 months. The median duration of response was 19.3 months in the phase III study CA184-024, which evaluated IPI 10 mg/kg in combination with dacarbazine in patients with unknown *BRAF* mutation status [4], and was 6.7 months with VEM in the BRIM-2 study [7]. The BOR was 36.8 % during VEM2, showing ongoing sensitivity to BRAF inhibition.

The landscape of therapeutic options for advanced melanoma continues to evolve rapidly. Combinations of BRAF plus MEK inhibitors (e.g., dabrafenib plus trametinib, as well as vemurafenib plus cobimetinib) have shown an overall response rate of 66–70 % in patients with *BRAF*-mutated advanced melanoma [20–22] and are now approved for this indication by the US FDA.

Several studies of PD-1 inhibition alone or in combination with IPI have included treatment-naïve patients with *BRAF*-mutated advanced melanoma who could have been eligible for our study. The ORR observed with PD-1 inhibition alone (i.e., nivolumab [NIVO] or pembrolizumab) is in the range of 33–37 %, while the combination of NIVO plus IPI elicited an ORR of 52–67 % [23–26]. NIVO and pembrolizumab as monotherapy and NIVO in combination with IPI are now approved treatments for advanced melanoma. It is important to keep the above data in mind to contextualize this study in current clinical practice.

Although a number of very effective agents are now available for advanced melanoma, the best dosing schedules, combinations or sequencing are still not known and remain the focus of active investigation. As an example, a phase II, randomized study (CheckMate 064) showed consistent improvement in efficacy outcomes with NIVO followed by IPI versus IPI followed by NIVO (e.g., confirmed ORR at week 25: 41.2 % versus 20.0 %, respectively) [27]. The pattern of AEs reported in CheckMate 064 was similar to that previously reported with either agent, alone or in combination, whereas the frequency of AEs was consistent with previous reports for NIVO plus IPI [23, 24].

The sequencing strategy of targeted therapy followed by immune-modulation, demonstrated in this study with VEM followed by IPI, serves as one of the links in our efforts to understand how best to use these agents. Additional understanding of the immune-modulatory effects of BRAF as well as MEK inhibition in the tumor microenvironment is warranted. MEK inhibition may influence the activity of certain subsets of effector lymphocytes. BRAF inhibition with VEM may also activate lymphocytes via the paradoxical activation of MAPK in *BRAF* wild-type cells and contribute not only to some of the side effects observed with the combination of immunotherapy, but also to improved antitumor activity and clinical outcomes. This study sets the stage for further investigation of BRAF plus MEK inhibition followed by PD-1 with or without CTLA-4 inhibition for select groups of patients who have high tumor burden or rapidly progressive symptomatic disease, which will be borne out in the phase III ECOG study (NCT02224781) currently underway to evaluate dabrafenib plus trametinib followed by IPI plus NIVO at progression compared with IPI plus NIVO followed by dabrafenib plus trametinib at progression in patients with BRAF V600-mutant advanced melanoma.

Regardless of recent developments exploring multiple sequential combinations, further investigation of the potential of BRAF inhibition combined with immune checkpoint inhibition, especially the PD-1 pathway, may be worthwhile. Our study showed that the sequential combination of VEM and IPI had manageable safety and that tumors remain sensitive to BRAF inhibition after progressing on immunotherapy with IPI.

## Conclusions

We show that VEM (960 mg twice daily for 6 weeks) followed by IPI 10 mg/kg has a manageable safety profile. Although the combination of BRAF plus MEK inhibition and NIVO plus IPI immune checkpoint inhibition is more commonly used today than VEM or IPI monotherapy to treat advanced melanoma, this study shows that IPI has efficacy after treatment with VEM in patients with *BRAF*-mutated melanoma and that tumors remain sensitive to VEM re-treatment after progressing on IPI. In addition, because VEM and IPI are the major drivers of the AE profile associated with combination regimens (e.g., AE profile of NIVO plus IPI is primarily driven by IPI), evaluation of each agent alone may be informative regarding the safety profile of combination therapy when used in a sequential regimen. The benefits/risks of BRAF inhibitors followed by immunotherapy should be evaluated further in light of continuing developments in treatment options for metastatic melanoma.

## Abbreviations

AE, adverse event; ALT, alanine aminotransferase; AST, aspartate aminotransferase; BID, twice daily; BOR, best overall response; CA, California; CI, confidence interval; CTLA-4, cytotoxic T-lymphocyte antigen 4; DOR, duration of response; DOSD, duration of stable disease; ECOG, Eastern Cooperative Oncology Group; FDA, Food and Drug Administration; GI, gastrointestinal; HR, hazard ratio; IPI, ipilimumab; KM, Kaplan-Meier; mo, month; mWHO, modified World Health Organization; NCI CTCAE, National Cancer Institute Common Terminology Criteria for Adverse Events; NE, not evaluable; NIVO, nivolumab; NY, New York; ORR, objective response rate; OS, overall survival; PD, progressive disease; PD-1, programmed death receptor-1; PFS, progression-free survival; PO, by mouth; Q12W, every 12 weeks; Q3W, every 3 weeks; SAE, serious adverse event; SD, standard deviation; USA, United States of America; VEM, vemurafenib; VEM1-IPI, vemurafenib followed by ipilimumab treatment; VEM2, vemurafenib re-treatment

## References

[1] Hodi FS, O’Day SJ, McDermott DF, Weber RW, Sosman JA, Haanen JB (2010). Improved survival with ipilimumab in patients with metastatic melanoma. N Engl J Med.

[2] Lebbé C, Weber JS, Maio M, Neyns B, Harmankaya K, Hamid O (2014). Survival follow-up and ipilimumab retreatment of patients with advanced melanoma who received ipilimumab in prior phase II studies. Ann Oncol.

[3] Maio M, Grob JJ, Aamdal S, Bondarenko I, Robert C, Thomas L (2015). Five-year survival rates for treatment-naïve patients with advanced melanoma who received ipilimumab plus dacarbazine in a phase III trial. J Clin Oncol.

[4] Robert C, Thomas L, Bondarenko I, O’Day S, Weber J, Garbe C (2011). Ipilimumab plus dacarbazine for previously untreated metastatic melanoma. N Engl J Med.

[5] Schadendorf D, Hodi FS, Robert C, Weber JS, Margolin K, Hamid O (2015). Pooled analysis of long-term survival data from phase II and phase III trials of ipilimumab in unresectable or metastatic melanoma. J Clin Oncol.

[6] Chapman PB, Hauschild A, Robert C, Haanen JB, Ascierto P, Larkin J (2011). Improved survival with vemurafenib in melanoma with BRAF V600E mutation. N Engl J Med.

[7] Sosman JA, Kim KB, Schuchter L, Gonzalez R, Pavlick AC, Weber JS (2012). Survival in BRAF V600-mutant advanced melanoma treated with vemurafenib. N Engl J Med.

[8] Spagnolo F, Ghiorzo P, Queirolo P (2014). Overcoming resistance to BRAF inhibition in BRAF-mutated metastatic melanoma. Oncotarget.

[9] Wagle N, Emery C, Berger MF, Davis MJ, Sawyer A, Pochanard P (2011). Dissecting therapeutic resistance to RAF inhibition in melanoma by tumor genomic profiling. J Clin Oncol.

[10] Luke JJ (2015). Is there an optimal intersection for targeted and immunotherapy treatments for melanoma?. Am J Hematol Oncol.

[11] Hong DS, Vence L, Falchook G, Radvanyi LG, Liu C, Goodman V (2012). BRAF(V600) inhibitor GSK2118436 targeted inhibition of mutant BRAF in cancer patients does not impair overall immune competency. Clin Cancer Res.

[12] Boni A, Cogdill AP, Dang P, Udayakumar D, Njauw CN, Sloss CM (2010). Selective BRAFV600E inhibition enhances T-cell recognition of melanoma without affecting lymphocyte function. Cancer Res.

[13] Ott PA, Henry T, Baranda SJ, Frleta D, Manches O, Bogunovic D (2013). Inhibition of both BRAF and MEK in BRAF(V600E) mutant melanoma restores compromised dendritic cell (DC) function while having differential direct effects on DC properties. Cancer Immunol Immunother.

[14] Ribas A, Hodi FS, Callahan M, Konto C, Wolchok J (2013). Hepatotoxicity with combination of vemurafenib and ipilimumab. N Engl J Med.

[15] Wolchok JD, Neyns B, Linette G, Negrier S, Lutzky J, Thomas L (2010). Ipilimumab monotherapy in patients with pretreated advanced melanoma: a randomised, double-blind, multicentre, phase 2, dose-ranging study. Lancet Oncol.

[16] O’Day SJ, Maio M, Chiarion-Sileni V, Gajewski TF, Pehamberger H, Bondarenko IN (2010). Efficacy and safety of ipilimumab monotherapy in patients with pretreated advanced melanoma: a multicenter single-arm phase II study. Ann Oncol.

[17] Ratushny V, Gober MD, Hick R, Ridky TW, Seykora JT (2012). From keratinocyte to cancer: the pathogenesis and modeling of cutaneous squamous cell carcinoma. J Clin Invest.

[18] Wolchok JD, Hoos A, O’Day S, Weber JS, Hamid O, Lebbé C (2009). Guidelines for the evaluation of immune therapy activity in solid tumors: immune-related response criteria. Clin Cancer Res.

[19] Chapman PB, Hauschild A, Robert C, Larkin JMG, Haanen JBA, Ribas A, et al. Updated overall survival (OS) results for BRIM-3, a phase III randomized, open-label, multicenter trial comparing BRAF inhibitor vemurafenib (vem) with dacarbazine (DTIC) in previously untreated patients with BRAFV600E-mutated melanoma. J Clin Oncol. 2012;30(Suppl). abstract 8502.

[20] Long GV, Stroyakovskiy D, Gogas H, Levchenko E, de Braud F, Larkin J (2015). Dabrafenib and trametinib versus dabrafenib and placebo for Val600 BRAF-mutant melanoma: a multicentre, double-blind, phase 3 randomised controlled trial. Lancet.

[21] Robert C, Karaszewska B, Schachter J, Rutkowski P, Mackiewicz A, Stroyakovskiy D, et al. Two year estimate of overall survival in COMBI-v, a randomized, open-label, phase III study comparing the combination of dabrafenib and trametinib with vemurafenib as first-line therapy in patients with unresectable or metastatic BRAF V600E/K mutation-positive cutaneous melanoma. Presented at the European Cancer Congress; Vienna, Austria; September 28, 2015: abstract 3301.

[22] Larkin J, Yan Y, McArthur GA, Ascierto PA, Liszkay G, Maio M, et al. Update of progression-free survival (PFS) and correlative biomarker analysis from coBRIM: Phase III study of cobimetinib (cobi) plus vemurafenib (vem) in advanced *BRAF*-mutated melanoma. J Clin Oncol. 2015;33(Suppl): abstract 9006.

[23] Postow MA, Chesney J, Pavlick AC, Robert C, Grossmann K, McDermott D (2015). Nivolumab and ipilimumab versus ipilimumab in untreated melanoma. N Engl J Med.

[24] Larkin J, Chiarion-Sileni V, Gonzalez R, Grob JJ, Cowey CL, Lao CD (2015). Combined nivolumab and ipilimumab or monotherapy in untreated melanoma. N Engl J Med.

[25] Larkin J, Chiarion-Sileni V, Gonzalez R, Grob JJ, Cowey CL, Lao CD, et al. Efficacy and safety in key patient subgroups of nivolumab alone or combined with ipilimumab versus ipilimumab alone in treatment-naïve patients with advanced melanoma (CheckMate 067). Presented at the 40th Annual Meeting of the European Society for Medical Oncology; Vienna, Austria; September 25–29, 2015: abstract 3303.

[26] Robert C, Schachter J, Long GV, Arance A, Grob JJ (2015). Mortier L, et al, and KEYNOTE-006 investigators: pembrolizumab versus ipilimumab in advanced melanoma. N Engl J Med.

[27] Weber JS, Gibney G, Sullivan RJ, Sosman JA, Slingluff CL Jr, Lawrence DP, et al. Sequential administration of nivolumab and ipilimumab with a planned switch in patients with advanced melanoma (CheckMate 064): an open-label, randomised, phase 2 trial. Lancet Oncol. 2016; doi:10.1016/S1470-2045(16)30126-7. 10.1016/S1470-2045(16)30126-7 PMC5474305 27269740

